# Biochemical mechanism and biological effects of the inhibition of silent information regulator 1 (SIRT1) by EX-527 (SEN0014196 or selisistat)

**DOI:** 10.1080/14756366.2020.1758691

**Published:** 2020-05-05

**Authors:** Sylvain Broussy, Hanna Laaroussi, Michel Vidal

**Affiliations:** a Université de Paris, Faculté de Pharmacie de Paris, CiTCoM, 8038 CNRS, U 1268 INSERM, Paris, France; b Service biologie du médicament, toxicologie, AP-HP, Hôpital Cochin, Paris, France

**Keywords:** SIRT1, EX-527, enzyme inhibition, cell-based and *in vivo* biological assays

## Abstract

The human sirtuin silent information regulator 1 (SIRT1) is a NAD^+^-dependent deacetylase enzyme. It deacetylates many protein substrates, including histones and transcription factors, thereby controlling many physiological and pathological processes. Several synthetic inhibitors and activators of SIRT1 have been developed, and some therapeutic applications have been explored. The indole EX-527 and its derivatives are among the most potent and selective SIRT1 inhibitors. EX-527 has been often used as a pharmacological tool to explore the effect of SIRT1 inhibition in various cell types. Its therapeutic potential has, therefore, been evaluated in animal models for several pathologies, including cancer. It has also been tested in phase II clinical trial for the treatment of Huntington’s disease (HD). In this review, we will provide an overview of the literature on EX-527, including its mechanism of inhibition and biological studies.

## 1. Introduction

Human silent information regulator 1 (SIRT1) belongs to the sirtuin family of enzymes, which constitute class III of the histone deacetylase family (HDAC). It is the most studied of the seven human sirtuins known to date. It is a NAD^+^-dependent deacetylase, which deacetylates many protein substrates, including histones and transcription factors^1^. SIRT1 has been linked to type 2 diabetes^2^, cancer^3^, Alzheimer disease^4^, and more generally diseases of ageing^5^^,^^6^. In particular, the contradictory roles of human SIRT1 in cancer have been reviewed and are still a subject of debate^7^^,^^8^. To study these biological activities, the modulation of SIRT1 expression and activity by bioengineering (mutations, overexpression, siRNA, or knockout for example) has been largely employed^7^^,^^9^^,^^10^.

In addition to these genetic manipulation studies, pharmacological modulation of SIRT1 has been the subject of intense research. SIRT1 modulators in general and their roles in cancer in particular have been often reviewed, usually giving an overview of several inhibitors and activators, but limited information on each one^11–14^. We present here an overview of the literature data on the SIRT1 selective and potent inhibitor EX-527 (SEN0014196 or selisistat) since its first disclosure in 2005^15^. Key data are reported, regarding its mechanism of inhibition and inhibitory potency *in vitro*, its effect on various cell types (used alone or in combination with other molecules), biological studies in animal models, and results of a clinical trial. This review primarily describes studies in which EX-527 is the main compound of interest, but we also included selected studies using EX-527 as a control and/or pharmacological tool to explore SIRT1 related pathways. To complete this overview, we also included some examples in which the inhibitor EX-527 was used to counteract the effects of other molecules, such as SIRT1 activators.

## 2. *In vitro* assays of EX-527 on isolated enzymes and mechanism of inhibition

### 2.1. Discovery, properties, IC_50_ values, and structure/activity relationship studies

**Figure 1.**
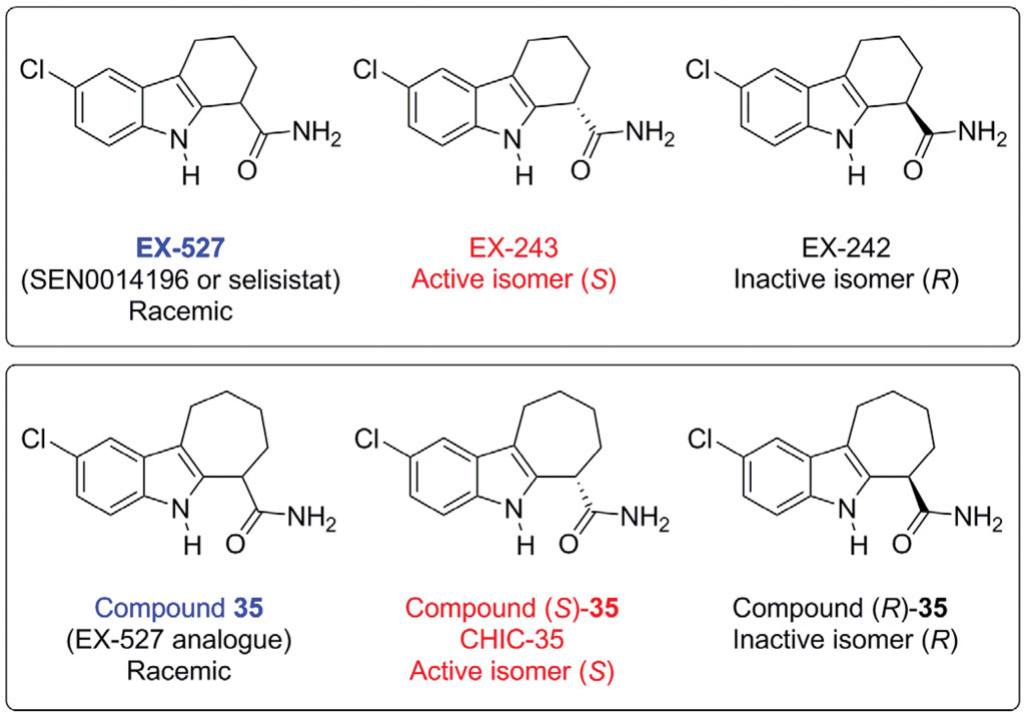
Structures of SIRT1 inhibitors EX-527 and its analogue Compound **35**, indicating their absolute stereochemistry and the corresponding names used in the literature^15^. EX-527 and CHIC-35 are now commercially available from suppliers.

EX-527 was identified in 2005 by high throughput screening of libraries of compounds on the enzyme SIRT1 (Figure 1)^15^. It has now been the subject of more than 200 articles.

**Scheme 1.**
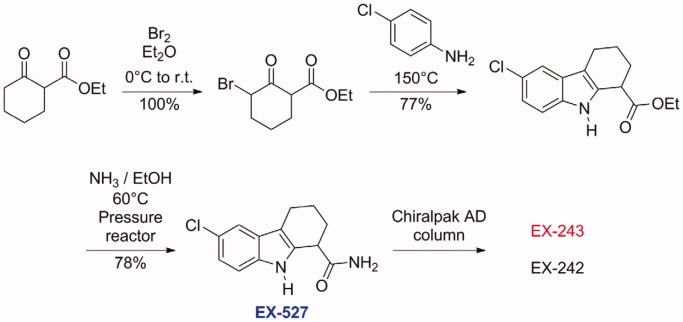
Chemical synthesis of EX-527^15^.

A typical synthesis of this family of compounds is depicted in Scheme 1. These compounds were obtained by a Bischler indole synthesis. In the first step, a β-keto ester was brominated on *α* to the ketone, affording a bromo keto ester, which was heated in the second step with an aniline, affording the tetrahydrocarbazole ester. The ester was then converted to the primary amide under pressure. In case enantiomerically pure material was needed, separation by chiral column chromatography was achieved^15^.

EX-527 is a potent and selective SIRT1 inhibitor, with IC_50_ values as low as 38 nM, depending on assay conditions^16^. In the first report, it was shown to be more selective for SIRT1 than for SIRT2 or SIRT3 (200–500-fold)^15^. EX-527 does not inhibit class I/II HDAC activity at concentrations up to 100 µM. EX-527 is racemic, the active isomer (designated EX-243) being (*S*), whereas the other (*R*) isomer (designated EX-242) is inactive. IC_50_ values for sirtuin inhibition by EX-527 have been measured in several studies, using a variety of assay methods and peptide substrates (Table 1). They range from 0.038 to 3 µM, usually between 0.1 and 1 µM. They depend mostly on the nature and concentration of the peptide substrates and on NAD^+^ concentration, because of the uncompetitive inhibition mechanism of EX-527 (see below). Very stringent structure/activity relationships were described in the original article^15^ and were later explained in light of the crystal structure published in 2013 (see below)^31^. Compound **35** (Figure 1) is an analogue of EX-527, very potent inhibitor of SIRT1: the IC_50_ of the (*S*) isomer is 60 nM, and the IC_50_ of the racemic mixture is 124 nM. It is selective for SIRT1, with an IC_50_ for SIRT2 of 2.77 µM^15^.

**Table 1.** *In vitro* assays of EX-527 and its analogue **35** on isolated recombinant sirtuins expressed in bacteria.

Compd	SIRT1	SIRT2	Other sirtuins	References
EX-527	0.098^a^	19.6^a^	SIRT3: 48.7^a^	Napper et al.^15^^,^^c^
	1.29^b^			
	0.038^a^^,^^d^		SIRT5: > 50 µM^e^	Solomon et al.^16^
	3 [1–5]^f^	79 [45–140]^g^		Huhtiniemi et al.^17^
	0.165 ± 0.050^h^			Liu et al.^18^
	0.125 ± 0.021^i^			
	0.74 ± 0.25^j^			Smith et al.^19^
	1.18 ± 0.24^k^			
	0.38^a^	32.6^l^		Peck et al.^20^
	0.16 ± 0.01^a^	> 10 (∼35% at 0.5 µM)^l^		Pasco et al.^21^
	0.16 ± 0.01^a^	48.5 ± 15.2^l^		Rotili et al.^22^
	83.6 ± 4.2% at 50 µM^a^	45.5 ± 2.8% at 50 µM^l^		Mellini et al.^23^
	0.26^m^	2.9^m^	SIRT3: > 50^m^	Disch et al.^24^
	0.09 ± 0.03^n^		SIRT3: 22.4 ± 2.7^o^	Gertz et al.^25^
			Sir2Tm: 0.90 ± 0.30^n^^,^^p^	
			SIRT5: > 25 µM^q^	
			SIRT6: 56 ± 8% at 200 µM^r^	Kokkonen et al.^26^
	0.33 ± 0.03^s^			Yang et al.^27^
	0.5^t^	6.5^t^		Therrien et al.^28^
	0.10 [0.05–0.19]^u^	3.0 [2.1–4.4]^u^	SIRT3: 165 [63–430]^u^	Ekblad et al.^29^
			SIRT6: 107 [48–240]^u^	
	0.1 ± 0.06^a^	20.1 ± 4.2^a^		Schnekenburger et al.^30^
EX-243	0.123^a^			Napper et al.^15^
EX-242	> 100^a^			
*Rac*-**35**	0.124^a^	2.77^a^	SIRT3: > 100^a^	
	0.652^b^			
(*S*)-**35**	0.063^a^			
(*R*)-**35**	23.0^a^			

IC_50_ values are given in µM (with errors as published) and/or %inhibition is indicated at the given concentration. This table constitutes an overview of representative data in the literature. It is important to note that only IC_50_ values from assays performed under the same experimental conditions are comparable.

^a^ Fluorimetric assay using a peptide substrate derived from the sequence of p53 (K382): Ac-RHKK(Ac)-AMC (AMC = 7-amino-4-methyl-coumarin).

^b^ Radioactive nicotinamide release assay using unlabelled 19-aminoacid peptide substrate.

^c^ SEM (standard error of the mean) < 30% for all data in this article.

^d^ SIRT1 expressed and purified from mammalian cells.

^e^ Release of [^3^H]acetate from acetylated cytochrome *c*.

^f^ Radioactive nicotinamide release assay using a peptide substrate derived from the sequence of p53 (K382): Ac-RHKK(Ac)-AMC.

^g^ Radioactive nicotinamide release assay using a peptide substrate derived from the sequence of p53 (K330): Ac-QPKK(Ac)-AMC.

^h^ Microfluidic mobility shift assay using a labelled peptide substrate derived from the sequence of p53 (K382): fluorescein-SKKGQSTSRHKK(Ac)LMFKTEGPDS.

^i^ NAD^+^ bioluminescence assay using a peptide substrate derived from the sequence of p53 (K382): HLKSKKGQSTSRHKK(Ac)LMFK.

^j^ Enzyme-coupled system detecting nicotinamide formation, using a peptide substrate derived from the sequence of histone H3 (K14) named AcH3: KSTGGK(Ac)APRKQ.

^k^ Charcoal-binding assay using [^3^H]AcH3.

^l^ Fluorimetric assay using a peptide substrate derived from the sequence of p53 (K330): Ac-QPKK(Ac)-AMC.

^m^ Mass spectrometry assay using the peptide substrate derived from the sequence of p53 (K382): Ac-RHKK(Ac)W-NH_2_.

^n^ Enzyme-coupled system detecting nicotinamide formation, using a peptide substrate derived from the sequence of p53 (K382): RHKK(Ac)LMFK.

^o^ Enzyme-coupled system detecting nicotinamide formation, using a peptide substrate derived from the sequence of acetyl-CoA synthetase 2 (ACS2, K642): TRSGK(Ac)VMRRL.

^p^ Sir2Tm: sirtuin from *Thermotoga maritima*.

^q^ Enzyme-coupled system detecting nicotinamide formation, using a peptide substrate derived from the sequence of carbamoyl phosphate synthetase 1 (CPS1, K527): FKRGVLK(Ac)EYGVKV.

^r^ Fluorimetric assay using a peptide substrate derived from the sequence of histone H3 (K56): Ac-RYQK(Ac)-AMC.

^s^ Luminescence assay using a peptide substrate derived from the sequence of p53 (K330): Z-QPK(Me)_2_K(Ac)-aminoluciferin.

^t^ Fluorometric assay using the substrate Cbz-K(Ac)-AMC.

^u^ Fluorometric assay kits, undisclosed substrates.

**Scheme 2.**
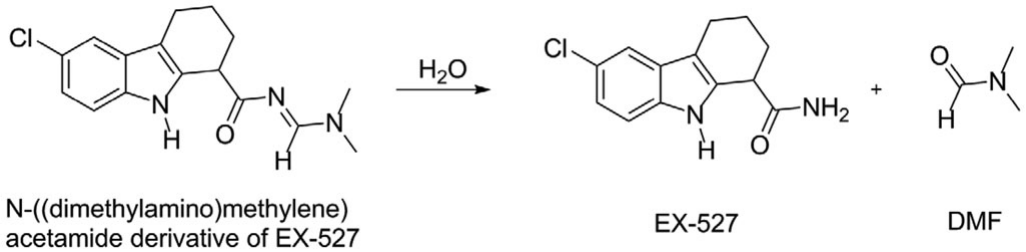
Spontaneous hydrolysis of the DMF adduct of EX-527.

EX-527 was also identified independently in 2006 from another high throughput screening. The screened compound was in fact the *N*-((dimethylamino)methylene)acetamide derivative (a dimethylformamide adduct), which was rapidly hydrolysed in aqueous solution to form EX-527 and dimethylformamide (Scheme 2)^32^.

EX-527 is also able to block the protein-protein interaction taking place between deleted in breast cancer 1 (DBC1) and SIRT1^33^. DBC1 is an endogenous protein shown to interact with SIRT1 and to inhibit its catalytic activity^34^^,^^35^. The regulation of this interaction is complex. For example, DBC1 itself is a substrate of SIRT1, and deacetylated DBC1 does not bind to SIRT1^33^. However, the team of Sinclair showed that EX-527 blocks the interaction *via* an acetylation-independent mechanism *in vitro*. They also demonstrated, using a luciferase complementation assay, that the inhibitor is able to block the SIRT1-DBC1 interaction in cells with an IC_50_ of approximately 1 µM^33^.

In addition to sirtuins, EX-527 and racemic **35** (*rac*-**35**) have been tested *in vitro* on other isolated enzyme and receptor targets. Overall, they displayed very little to no activity. They did not inhibit class I and II HDACs and NAD^+^ glycohydrolase at 100 µM^15^. PARP are enzymes using the NAD^+^ as cosubstrate for ADP-ribosyl transfer, producing nicotinamide, like sirtuins. Therefore, inhibitors targeting the nicotinamide binding pocket like EX-527 could have an inhibitory effect on PARP enzymes. No inhibition was observed on PARP1 and PARP10^29^^,^^36^. On cardiac potassium channels (hERG/I_Kr_), EX-527 had an IC_50_ of 43 µM, with 0% inhibition at 10 µM^37^, and *rac*-**35** displayed only 10% inhibition at 10 µM^15^. Cytochrome P450 are key enzymes involved in metabolism of drugs. They are largely evaluated in screening panels of new biologically active molecules, to identify P450 substrates or inhibitors. On cytochromes P450 (3A4, 2D6, 2C9, 2C19, 1A2, 2C8, and 2E1), both molecules had weak or no inhibitory potency at 1 µM, the highest values being 23% inhibition for 2C19 and 1A2 with *rac*-**35**. IC_50_ values determined for EX-527 were higher than 100 µM for all cytochromes P450 except 2C9 (62.4 µM), 2C19 (72.2 µM), and A2 (8.7 µM)^15^^,^^37^.

### 2.2. Mechanism of inhibition and crystal structures

**Figure 2.**
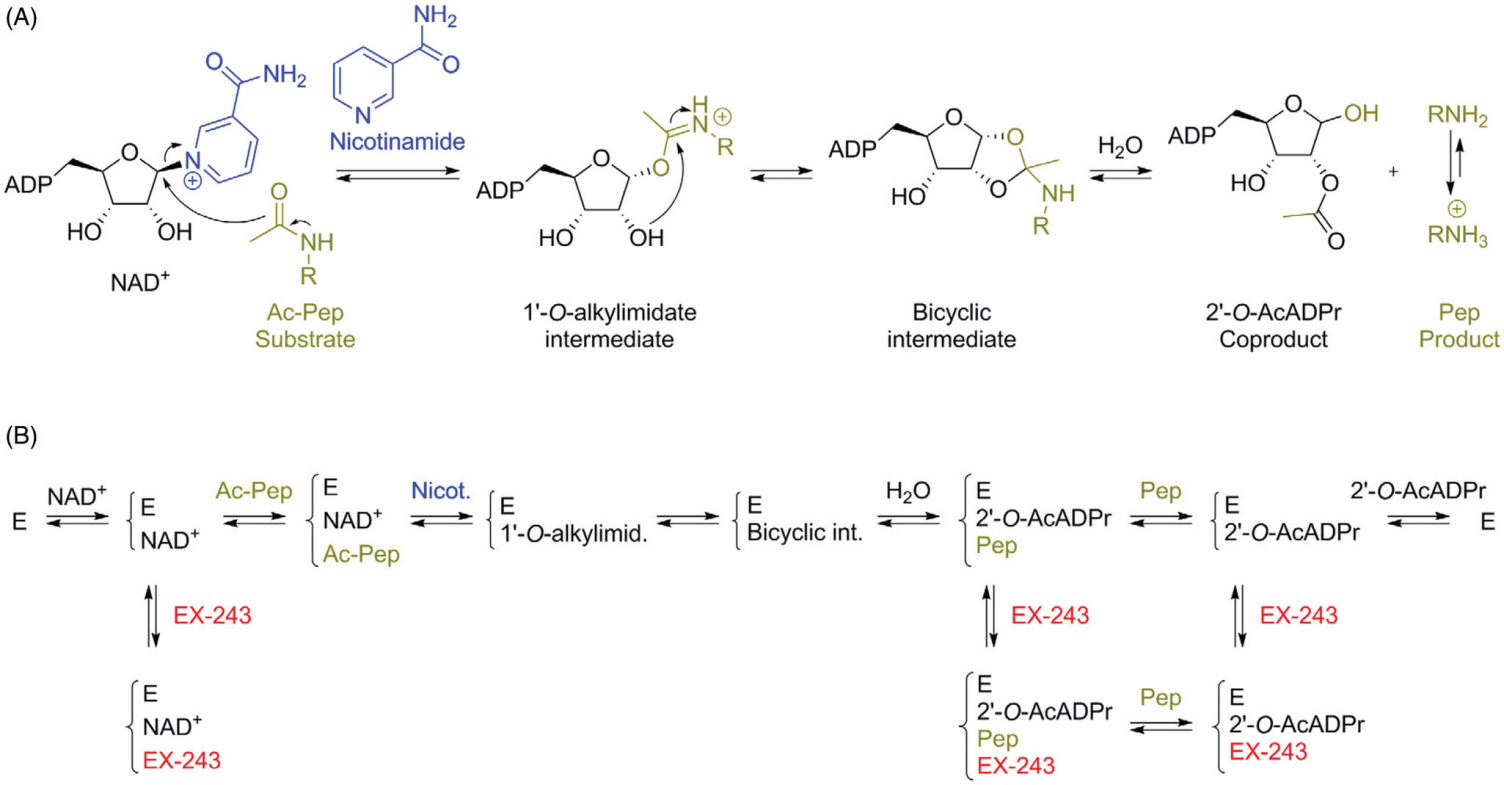
(A) Mechanism of sirtuin-catalysed deacetylation of a peptide (or protein) substrate Ac-Pep (acetylated peptide). For simplicity, acidic and basic general catalysis is not represented in this mechanism. (B) Proposed simplified mechanism of sirtuin inhibition by EX-243, adapted from Gertz et al.^25^. E: enzyme. Note that former studies of SIRT1 inhibition by substrate analogues suggested (i) a random addition of substrates (therefore, Ac-Pep could be added first to the enzyme, not represented here for simplification) and (ii) a departure of the peptide product from the enzyme in the last step (which would disagree here with the existence of the crystallised complex E/2′-*O*-AcADPr/EX-243)^39^.

A simplified mechanism of deacetylation of a substrate catalysed by sirtuins is represented in Figure 2(A)^38^. The acetylated substrate makes a nucleophilic substitution on the C1′ of the NAD^+^ cofactor, releasing nicotinamide. The 1′-*O*-alkylimidate intermediate formed reacts intramolecularly to generate a bicyclic intermediate. This intermediate is subsequently hydrolysed to form the deacetylated product and the 2′-*O*-AcADPr coproduct.

The mechanism of SIRT1 inhibition by EX-527 is represented in Figure 2(B), adapted from Gertz et al.^25^. Mechanistic studies on SIRT1, SIRT3, and Sir2Tm (sirtuin from *Thermotoga maritima*) demonstrated in all three cases that the inhibition by EX-527 was non-competitive with substrate and uncompetitive with NAD^+^. Therefore, the inhibition potency depends on the NAD^+^ concentration. Binding parameters are summarised in Table 2. K_d_ values for EX-527 measured for the apo enzymes and in the presence of NAD^+^ confirmed the uncompetitive nature of the inhibition. Indeed, EX-527 does not bind to the apo enzyme, but binds with low micromolar affinity in the presence of NAD^+^.

**Table 2.** Binding parameters of EX-527 with sirtuins.

Sirtuin	K_i_ (Ac-Pep)	K_i_ (NAD^+^)	K_d_ (apo)	K_d_ [Ac-Pep]	K_d_ [NAD^+^]	K_d_ (Ac-Pep + NAD^+^)	References
SIRT1	0.408^a^ Mixed	0.287^a^ Mixed/uncompetitive	Not binding^b^		1.3^b^ [1 mM]		Napper et al.^15^ Zhao et al.^31^
Sir2Tm	1.8 ± 0.4^c^ Non-competitive	3.3 ± 0.4^c^ Uncompetitive	>180^e^	>170^e^ [1 mM]	6.0 ± 0.4^e^ [5 mM]	4.9 ± 0.5^e^	Gertz et al.^25^
SIRT3	33.4 ± 4.4^d^ Non-competitive	31.3 ± 2.1^d^ Uncompetitive	>330^e^	>180^e^ [1 mM]	16.5 ± 2.9^e^ [5 mM]	10.0 ± 1.4^e^	

K_i_ and K_d_ values are given in µM (Ac-pep: acetylated peptide).

^a^ Fluorimetric assay using a peptide substrate derived from the sequence of p53 (K382): Ac-RHKK(Ac)-AMC (AMC = 7-amino-4-methyl-coumarin).

^b^ Determined by SPR.

^c^ Enzyme-coupled system detecting nicotinamide formation, using a peptide substrate derived from the sequence of p53 (K382): RHKK(Ac)LMFK.

^d^ Enzyme-coupled system detecting nicotinamide formation, using a peptide substrate derived from the sequence of acetyl-CoA synthetase 2 (ACS2, K642): TRSGK(Ac)VMRRL.

^e^ K_d_ values determined using microscale thermophoresis.

Another interesting aspect of these mechanistic studies concerns the specificity of EX-527 for sirtuin isoforms. The authors propose that the difference between EX-527-sensitive enzymes (like SIRT1 and Sir2Tm) and less sensitive ones (like SIRT2 and SIRT3) comes from differences in their kinetics of catalysis, and not from differences in the binding pockets, which are very similar^25^. Indeed, they suggest that binding of EX-527 either after or before the rate-limiting step leads to differences in inhibition potency.

Six crystal structures of sirtuins in complex with the active enantiomers of the inhibitors, compound (*S*)-**35** and EX-243, have been described. The first one was between SIRT1, NAD^+^, and compound (*S*)-**35** (PDB 4I5I)^31^. The others were part of a series of structures solved to study the mechanism of sirtuin inhibition by EX-243 (the active enantiomer of EX-527), with human SIRT3 and Sir2Tm: SIRT3/NAD^+^/EX-243 (4BV3), SIRT3/ADPr/EX-243 (4BVB), SIRT3/2′-*O*-AcADPr/EX-243 (4BVH), Sir2Tm soaking (4BUZ, partially with substrates Ac-p53 peptide and NAD^+^, and partially with product 2′-*O*-AcADPr and EX-243), and Sir2Tm/2′-*O*-AcADPr/deacetyl p53 peptide/EX-243 (4BV2)^25^.

**Figure 3.**
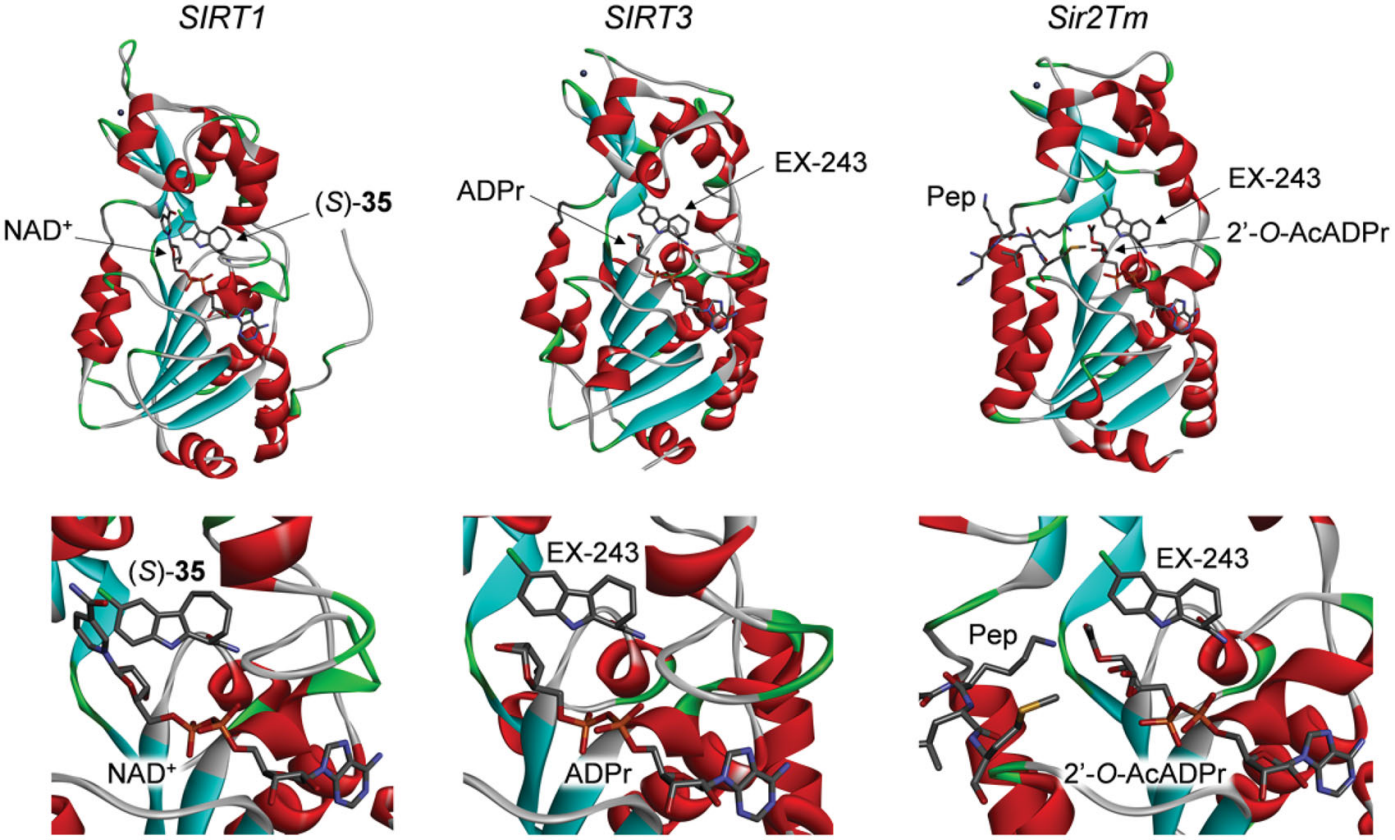
Crystal structures of sirtuins in complex with indole inhibitors EX-243 and its analogue (*S*)-**35**. Left: SIRT1/NAD^+^/(*S*)-**35** (4I5I)^31^; middle: SIRT3/ADPr/EX-243 (4BVB); right: Sir2Tm/2′-*O*-AcADPr/deacetyl p53 peptide/EX-243 (4BV2)^25^. Active site close-up representations are displayed below the full structures. Pep: deacetyl p53 peptide.

In all these structures, the inhibitors occupy the nicotinamide binding pocket (the so-called C-pocket) of the sirtuin, and one of the following molecules is also co-crystallised, forming a ternary complex: NAD^+^, the coproduct 2′-*O*-AcADPr, or ADPr (Figure 3). This observation is in agreement with the uncompetitive nature of the inhibition with the cofactor NAD^+^, which is required for efficient inhibition, as mentioned above. The inhibitors are deeply buried in the C-pocket and make hydrogen bonds contacts and hydrophobic interactions with the enzyme, which explain the stringent structure/activity relationships observed^15^.

Moreover, the mechanistic studies showed that sirtuin inhibition with EX-527 allows the formation of one molecule of product per molecule of enzyme, indicating that the inhibitor binds most efficiently after bicyclic intermediate formation and allows coproduct formation^25^. The authors proposed that EX-243 inhibits sirtuins mostly by binding in the presence of the coproduct 2′-*O*-AcADPr. Finally, from the comparison of crystals structures with and without the inhibitor, it appears that a flexible cofactor-binding loop moves towards the inhibitor and the coproduct during inhibition, resulting in a “closed” conformation preventing product release^25^.

## 3. Cellular assays of EX-527

EX-527 has been tested on several cell lines, either as the main molecule of interest for potential therapeutic applications, or as a control experiment for comparison with other sirtuin modulators (inhibitors or activators). Often, it has been used as a pharmacological tool to demonstrate the involvement of SIRT1 in a biological response. An overview of literature data is summarised in Table 3.

**Table 3.** Representative examples of cellular effects of EX-527.

Cell lines^a^	Added agent	Effect of EX-527 on cells	Effect of EX-527 at the protein level	Comments	References
NCI-H460 U-2 OS MCF-7 HMEC	Etoposide, adriamycin, hydroxyurea, or hydrogen peroxide	No effect at 1 µM	Increases p53 acetylation (K382) at 1 µM (but no effect on two specific p53 target genes)	No effect on p53 without the genotoxic agent − 1 µM is non-toxic to all cell lines	Solomon et al.^16^
HCT-116	5-FU or camptothecin	Decreases cell proliferation and increases apoptosis at 2 µM	–	Increases cell proliferation at 2 µM, *without the chemotherapy agent* (and under growth factor deprivation)	Kabra et al.^40^
MCF-7	None	Decreases proliferation at 50–100 µM	No apparent increase in p53 acetylation, but global increase in lysine acetylation of proteins	Causes cell cycle arrest at G1 phase at 50 µM	Peck et al.^20^
U937	None	No cytotoxicity up to 50 µM ∼10 % apoptosis induction at 50 µM	–	No effect on granulocytic differentiation at 50 µM	Rotili at al. ^22^
Primary AML Primary B-CLL U937 697 Jurkat	Valproic acid (VA): HDAC class I/II/IV inhibitor	Synergistic effect with VA (100 µg/mL): ∼60% leukaemia cell death at 75 µM	Effect through Bax: in Jurkat with increased Bax expression, ∼70% leukaemia cell death at 75 µM (even without VA)	Low cytotoxic activity in leukaemia cells without VA	Cea et al.^41^
SGC transfected with ATF4 (induces MDR effects)	5-FU or cisplatin	Increases the cytotoxicity of 5-FU and cisplatin at 10 µM (synergistic effect)	Downregulates MDR1 expression	Slightly increases the viability at 10 µM without the cytotoxic agent	Zhu et al.^42^
MCF-7 U937	None	Cell cycle arrest in the G1 phase (no apoptosis) at 50 µM	At 10 µM, increases p53 and α-tubulin acetylation	No effect on granulocytic differentiation at 50 µM	Mellini et al.^23^
CSC: CRC (CRO and 1.1) GBM (30P and 30PT)	None	Weak inhibition of cell viability at 50 µM (up to 20%)	–	In combination with SIRT2 inhibitor AGK2, slight synergic effect proposed	Rotili et al.^43^
HCT-116	None	–	At 10 µM, increases p53 acetylation	Ratio (Ac-p53 / total p53) = 0.27 vs control = 0.03	Suzuki et al.^44^
BMDMs	LPS-induced production of cytokines	At 4 µM, no effect on cytokine production by macrophages	–	No effect at 120 µM or in combination with SIRT2-selective inhibitors	Lugrin et al.^45^
HCC (HepG2)	Trichostatin (TSA): HDAC inhibitor	–	At 20 µM: increases p53 acetylation decreases NAMPT enzymatic activity and increases its extracellular levels	–	Schuster et al.^46^
PC-12 expressing mHtt	None	Rescues ∼35% mHtt mediated toxicity at 1 µM (but only ∼25% at 10 µM)	Increases mHtt acetylation and clearance	Protective effect in primary cultures of rat striatal neurons infected with viral vectors expressing a mHtt fragment	Smith et al.^47^
SH-SY5Y	None	At 3 µM, restores viability in neuronal cells carrying a G93A SOD1 mutant (ALS-linked mutation)	No increase in p53 acetylation	The authors propose that the observed effects do not come from SIRT1 inhibition	Valle et al.^48^
HUVEC	H_2_O_2_	At 15 µM, protects against H_2_O_2_: Increases cell viability, adhesion, migratory ability Decreases the apoptotic index and ROS production	Reverses H_2_O_2_ effects: Decreases SIRT1, p-JNK, p-p38MAPK and increases p-ERK expression	No effect on HUVEC untreated by H_2_O_2_	Li et al.^49^
PANC-1 BXPC-3 ASPC-1	Gemcitabine or cisplatin	At 1 µM, increases the cytotoxic and pro-apoptotic effects of gemcitabine and cisplatin	At 2 µM, increases p53 acetylation and FOXO3a expression	Pro-apoptotic and anti-proliferative effects also without the cytotoxic agent (IC_50_ values 5 to 9 µM)	Zhang et al.^50^
TNBC MDA-MB-231 BT-549	None	Decreases viability by 20% at 50 µM	At 25 µM, increases p53 acetylation (K382)	Additional complex interplay with AMPK and metadherin studied	Gollavilli et al.^51^
CSCs: CD44^high^ CML K562 CD44^+^ HCT-15	Hsp90 inhibitors: 17-AAG and AUY922	At 10 nM, increases the cytotoxicity of Hsp90 inhibitors	Involvement of HSF1 and MDR related molecules proposed	–	Kim et al.^52^
CEM/VLB_100_ MCF7-MDR (MDR variants)	Hsp90 inhibitors: 17-AAG and AUY922	At 10 nM, increases the cytotoxicity of Hsp90 inhibitors (synergistic effect demonstrated)	At 50 nM: Decreases 17-AAG induced expression of Hsp70/Hsp27 Increases 17-AAG induced downregulation of mut p53 and P-gp Decreases P-gp efflux activity with AUY922	Decreases P-gp efflux activity also without AUY922	Kim et al.^53^
HCC (HepG2)	H_2_O_2_	–	At 10 µM, aggravates H_2_O_2_ induced: Decrease in MnSOD and Bcl-xL Increase in cleaved caspase 3	–	Hu et al.^54^
HHUA, HHUA-SIRT1, HEC151 and HEC1B	Cisplatin	At 1 µM, inhibits the proliferation with a synergic effect with cisplatin	Independent of p53 mutation status	Inhibits the proliferation at 1 µM also without cisplatin	Asaka et al.^55^
Human platelets	None	At 10 µM, induces apoptosis-like changes: enhances annexin V binding, ROS production and drop in mitochondrial transmembrane potential	Increases p53 acetylation and the level of conformationally active Bax	–	Kumari et al.^56^
Naïve CD4 T cells	None	At 12.5 µM, decreases Th17 effector cells differentiation from CD4 T cells	SIRT1 deacetylates RORγt and increases its transcriptional activity	–	Lim et al.^57^
HeLa	None	At 10 µM, decreases colony formation (> 50 %) and migration At 50 µM, causes cell cycle arrest in the G1 phase (no apoptosis)	Increases HSF1 acetylation, ubiquitination, and degradation Causes G1 phase arrest mediated by inhibition of Cdk4, Cdk6 and cyclin D1	–	Kim et al.^58^
Pluripotent P19 cells (mouse embryonic carcinoma)	None	At 100 µM, accelerates the differentiation of P19 cells into functionally active neurons	Identification of neuron-specific proteins and glutamate receptor in differentiated neurons	–	Kim et al.^59^
A549	MK-1775: WEE1 kinase inhibitor (induces DNA damage)	At 5 µM, enhances the anti-proliferative and pro-apoptotic effects of MK-1775.	Reduces homologous recombination (HR) repair activity by acetylation of machinery proteins NBS1 and Rad51	Several other lung cancer cells lines tested give similar results	Chen et al.^60^
THP-1 macrophages	Ox-LDL induced inhibition of autophagy	At 2 µM, increases the inhibition of autophagy	Exacerbates acetylation of Atg5	Macrophage accumulation is linked to atherosclerosis	Yang et al.^61^
AML12 RAW264.7 macrophages	[Ru(CO)_3_Cl_2_]_2_ (Carbon monoxide releasing molecule)	At 10 µM, decreases the protective effect of [Ru(CO)_3_Cl_2_]_2_ after hypoxia/reoxygenation injury	Decreases the inhibition of acetylation, translocation to the cytoplasm, and release of HMGB1 by [Ru(CO)_3_Cl_2_]_2_	A direct deacetylation of HMGB1 by SIRT1 was also demonstrated with isolated enzymes	Sun et al.^62^
U373 Hs683	None	Inhibits cell growth with IC_50_ = 157.4 ± 23.0 (U373) and 115.9 ± 23.3 µM (Hs683)	–	–	Schnekenburger et al.^30^
HCC (HepG2 and Huh7)	None	Decreases cell survival with IC_50_ = 195 ± 12 (HepG2) and 33 ± 6 µM (Huh7) and increases early apoptosis at 1 µM	Increases p53 and FoxO1 acetylation at 1 µM Decreases ABC transporters P-gp and MRP3 protein levels at 40 µM in HepG2	3D cultures: decreases spheroid growth and viability with IC_50_ = 567 ± 41 (HepG2) and 67 ± 16 µM (Huh7)	Ceballos et al.^63^
T cells	None	At 50 µM, increases the number and the suppressive function of Tregs	Increases both the acetylation and the expression levels of FOXP3	T cells isolated from patients suffering from abdominal aortic aneurysm	Jiang et al.^64^
HCC (HepG2)	Hesperetin	At 10 µM, no effect on cell viability	Inhibits the increase of SIRT1 activity and AMPK phosphorylation caused by hesperetin	–	Shokri Afra et al.^65^
BMMs	RANKL-induced Osteoclastogenesis	Promotes RANKL-stimulated osteoclastogenesis	Increases TNF-α mRNA and protein levels and ROS production	Dose of EX-527 not found	Yan et al.^66^
HUVEC	High glucose conditions Resveratrol	At 10 µM, abolishes resveratrol-mediated anti-apoptosis and pro-proliferation effects	Involvement of the transcription factors Foxo1 and c-Myc	–	Huang et al.^67^
HL-7702	Isoniazid (antituberculosis drug)	At 1 µM, aggravates the cell damages caused by isoniazid	In combination with isoniazid, increases further the expression of inflammatory regulators and cytokines, and the level of H3K9 acetylation in the promoter region of the IL-6 gene	No effects on cells and proteins when used alone	Zhang et al.^68^
T cells stimulated with allogenic APC (co-cultures)	None	At 10 µg/mL, reduces T cell proliferation	Increases p53 acetylation and total protein acetylation	–	Daenthanasanmak et al.^69^
MDA-MB-231 (high NNMT expression)	Adriamycin or paclitaxel	Increases the cytotoxicity, the inhibition of colony formation, and the apoptosis caused by the cytotoxic agents	Decreases the protection against cytotoxic agents given by the high NNMT expression	No effect without a cytotoxic agent Dose of EX-527 not found	Wang et al.^70^

^a^ Cell lines: 697: B cell precursor leukaemia; A549: adenocarcinomic human alveolar basal epithelial cells (lung cancer); AML12: alpha mouse liver 12 (from hepatocytes); ASPC-1: pancreatic cancer; B-CLL: B cell chronic lymphocytic leukaemia; BM(D)Ms: bone-marrow derived macrophages; BXPC-3: pancreatic cancer; CEM/VLB_100_: MDR variant of acute lymphoblastic leukaemia cells (overexpressing P-gp); CML: human chronic leukaemia; CRC: colorectal cancer; CSCs: cancer stem-like cells; GBM: glioblastoma multiforme; HCC: hepatocellular carcinoma; HCT-116/HCT-15: human colon cancer; Hela: cervical cancer; HHUA, HEC151, and HEC1B: human endometrial carcinoma; HMEC: primary human mammary epithelial cells; HL-7702: human normal liver cells; Hs683: glioblastoma; HUVEC: human umbilical vein endothelial cells; Jurkat: acute T cell leukaemia; MCF-7: human breast cancer; MDA-MB-231: breast cancer; NCI-H460: human non-small cell lung cancer; PANC-1: pancreatic cancer; PC-12: rat pheochromocytoma cells; SGC7901: human gastric adenocarcinoma; SH-SY5Y: subclone from bone marrow cells from neuroblastoma; Th17: T helper 17 cells (not naïve CD4 T cells); THP-1: human leukaemia monocyte; TNBC: triple negative breast cancer; Tregs: T regulatory cells; U373: glioblastoma; U937: human myeloid leukaemia (AML: acute myelogenous leukaemia); U-2 OS: human bone osteosarcoma epithelial cells.

5-FU: 5-fluorouracil; ABC: ATP binding cassette; AMPK: AMP-activated protein kinase; APC: antigen-presenting cells; ATF4: activating transcription factor 4; Atg5: autophagy-related 5; Bcl-xL: B cell lymphoma-extra-large; FoxO: forkhead box O; FOXP3: human forkhead box P3; HMGB1: high-mobility group box 1; HSF1: heat shock factor 1; Hsp: heat shock protein; LPS: lipopolysaccharides; MRP3: multidrug resistance-associated protein 3; mHtt (mHttex1pQ72): mutated Htt (huntingtin) exon 1 fragment with expanded Q repeat, presenting aggregates, and cytotoxicity, model of Huntington’s disease (HD); MnSOD: manganese superoxide dismutase; NNMT: nicotinamide *N*-methyl transferase; Ox-LDL: oxidised low-density lipoprotein; P-gp/MDR1: P-glycoprotein/multidrug resistance protein 1; RANKL: receptor activator of nuclear factor-κB ligand; RORγt: RAR-related orphan receptor γ-t; TNF-α: tumour necrosis factor-α

On tumour cell lines, several reports demonstrated the ability of EX-527 to increase p53 acetylation from 1 to 25 µM concentrations, when used either alone or in combination with cytotoxic molecules^16^^,^^23^^,^^44^^,^^46^^,^^51^^,^^56^^,^^63^. EX-527 was shown to improve the efficiency of cytotoxic agents on cancer cells, with several chemotherapeutic and genotoxic agents^40^^,^^42^^,^^60^. However, in few cases, EX-527 administered alone *increased* cell proliferation of cancer cell lines^49^^,^^71^. The conclusion of one of these studies on the role of SIRT1 in cancer cells is a simple summary of these apparently contradictory results:

In summary, our results suggest that both activators and inhibitors of SirT1 have therapeutic potential as anti-tumor agents. A simple scenario is that SirT1 activators may impart cancer prevention effects by enhancing the growth-inhibitory effect of SirT1 in benign tumors. Its effect on advanced stage tumors may be heterogeneous, depending on whether a tumor has evolved to rely on SirT1 for survival. However, when tumors are being treated with chemotherapy, SirT1 inhibitors may be useful for enhancing apoptotic response^40^.

Ten years after this report, the list of EX-527 studies has grown to reinforce this view (Table 3). For example, a decrease in cell survival and migration and an increase in apoptosis was recently observed on hepatocellular carcinoma (HCC: HepG2 and Huh7) cell lines with EX-527 alone^63^. Moreover, the same study demonstrated that EX-527 induced the downregulation of ABC transporters P-gp and MRP3 in HepG2 cells, suggesting an additional potential application of this SIRT1 inhibitor in combination with conventional therapeutic drugs to overcome multi-drug resistance (MDR) during HCC therapy^63^. Indeed, one of the most potent effect was obtained when EX-527 was used in combination with Hsp-90 inhibitors on CSCs (cancer stem-like cells) or MDR variants, with a potent increase in cytotoxicity of the Hsp-90 inhibitor with only 10 nM EX-527^52^^,^^53^. Moreover, EX-527 at 1 µM decreased colony formation of ovarian carcinoma cells, with or without overexpression of SIRT1^72^. At 600 nM, it suppressed cell migration and inhibited the occurrence of epithelial–mesenchymal transition (EMT) in chemotherapy resistant oesophageal cancer cells^71^. Overall, several factors are important to consider to understand the effect of EX-527 on cancer cells: (i) the type of cell line and the cancer stage, from benign to advanced, (ii) the presence of other agents, conventional chemotherapy, or additional HDAC inhibitors for example, and (iii) the dose, because at higher doses (ex. 40 µM or above), EX-527 may significantly inhibit SIRT2 and may have other targets. For potential anti-cancer therapeutic applications, aiming for a specific SIRT1 inhibition at low concentrations of EX-527 (ex. 1 µM or below) in combination with cytotoxic agents may be the most promising strategy.

On non-cancer cell lines, fewer studies were published than on cancer-cell lines. For example on HUVEC, EX-527 was shown to protect from H_2_O_2_ damage^49^, but to abolish the protective effect of resveratrol under high-glucose conditions^67^. Several articles described effects on cells involved in the immune system, macrophages, and T cells. Beneficial effects on autoimmune diseases and graft rejection problems can be envisioned from these cell assays, for example through reduction of effector T cell proliferation and differentiation^57^^,^^69^, and increase in the number and suppressive function of T regulatory cells Tregs (see Chapter undefined for *in vivo* results)^64^.

Many of the studies evaluating the role of EX-527 in cells summarised in this review incorporated control experiments with SIRT1 knockdown, mostly with anti-SIRT1 siRNA. These studies, in which the same effects were obtained with anti-SIRT1 siRNA or with its pharmacological inhibition with EX-527, make a strong case for the use of EX-527 as a pharmacological tool to study SIRT1 activity. However, the fact that EX-527 only targets SIRT1 must be tempered. Indeed, *in vitro* studies show that the extent of its specificity, in particular towards SIRT2, depends on the assay types (nature of the substrate and concentration of NAD^+^ for example) and may not be so high under certain conditions (Table 1). Consequently, its specificity inside cells or *in vivo* is even less predictable and quantifiable. Therefore, the results of studies concluding that SIRT1 is involved in the observed effect must be taken with caution, if they are solely based on the effect of EX-527 as a pharmacological control. SIRT2 and other unknown potential protein targets may be involved.

## 4. *In vivo* assays of EX-527

EX-527 has been tested in several organisms, mostly mice and rats, but also in the nematode *C. elegans*, in *Drosophila melanogaster* (*D. melanogaster*) and in humans in exploratory clinical trials (Tables 4 and 5).

**Table 4.** Selected pharmacokinetics parameters of EX-527 (in plasma).

Organism	Dose	C_max_ (µM)	*t*_max_ (h)	*t*_1/2_ (h)	C_ss,avg_ (µM)	References
C57bl/6J mice	10 mg/kg			2.3		Napper et al.^15^
R6/2 mice (mean ± SD, *n* = 3)	5 mg/kg	6.9 ± 6.9	0.3 ± 0.1	2.7 ± 2.3	0.4 ± 0.2	Smith et al.^47^
	10 mg/kg	10.5 ± 3.6	0.8 ± 0.4	1.4 ± 0.5	1.5 ± 0.4	
	10 mg/kg^a^	21.5 ± 3.3^a^	1.0 ± 0.0^a^	2.8 ± 0.4^a^	3.0 ± 0.4^a^	
	20 mg/kg	29.3 ± 6.4	0.5 ± 0.0	0.9 ± 0.2	3.2 ± 0.4	
Healthy human volunteers^b^	150 mg	6.7 ± 1.8	3.7	3.9 ± 1.6	1.6 ± 0.6	Westerberg et al.^37^
	300 mg	13.1 ± 4.5	3.5	4.9 ± 0.8	3.9 ± 2.2	
	600 mg	26.6 ± 10.5	4.0	6.1 ± 1.4	11.8 ± 6.0	
HD patients^b^	10 mg/d	0.6 ± 0.2	2.0	2.3 ± 0.9	0.11 ± 0.05	Süssmuth et al.^73^
	100 mg/d	5.9 ± 1.9	3.0	3.3 ± 1.6	1.8 ± 0.9	

R6/2 is a mice model of Huntington’s disease (HD).

C_max_: maximal plasma concentration; *t*_1/2_: terminal plasma half-life; C_ss,avg_: average plasma concentration over 24 h.

^a^ Values measured in brain.

^b^ Data selected for males (larger samples and dose ranges).

**Table 5.** Representative examples of *in vivo* assays of EX-527.

Organism	Physiology/pathology	Effect of EX-527	Proposed protein(s) and/or pathway(s) involved	References
Transgenic nematodes *Caenorhabditis elegans*	Oculopharyngeal muscular dystrophy (OPMD)	Fully rescues motility at 33.3 µM	Sir2^a^ inhibition modulates the activity of FoxO transcription factor, therefore, decreasing polyalanine expansion in PABPN1	Pasco et al.^21^
Transgenic flies *Drosophila melanogaster*	Model of Huntington’s disease (HD)	At 0.1 and 1 µM, limits the loss of photoreceptor neurons At 10 µM, increases the survival of flies	Sir2^a^ inhibition increases acetylation of mHtt exon 1 fragment, increasing its rate of clearance. Beneficial effects were eliminated in Sir2 (−/−) flies	Smith et al.^47^
C57BL/6 mice	Heart allograft	At 1 mg/kg/d in combination with rapamycin, prolonged heart allograft survival	Involvement of Foxp3 in Tregs cells	Beier et al.^74^
Mice	Adoptively transferred Tregs (potential applications in autoimmune diseases and graft rejections)	At 40 mg/kg/d i.p., increases Tregs stability	Promotes Foxp3 expression in Tregs, by increasing acetylation on 3 of its lysine sites	Kwon et al.^75^
R6/2 mice	Model of HD	At 20 mg/kg, increases the median survival by 3 weeks and decreases the number of aggregates in brains At 5 mg/kg, reduces the ventricular volume in brains (but not significant at 20 mg/kg)	Increases acetylation of mHtt exon 1 fragment, increasing its rate of clearance Possibly other SIRT1 substrates involved	Smith et al.^47^
Mice	Thrombocytopenia	At 20 mg/kg, decreases the platelet count and the number of reticulated platelets	Increases the acetylation of p53 and the level of conformationally active Bax	Kumari et al.^56^
C57BL/6J mice	Sepsis induced by caecal ligation and puncture	At 5 mg/kg i.p., abolishes the protective effects of melatonin	FoxO1, p53, NF-κB, and Bax	Zhao et al.^76^
Mice	Model of multiple sclerosis	At 10 mg/kg subcutaneous injection, strongly suppresses the number of paralysed mice (from 100 to ∼20%)	Effect on Th17 effector cells through RORγt	Lim et al.^57^
Mice	Endometrial cancer model with HHUA and HEC1B cells xenografts	At 10 mg/kg/week i.p.:Decreases the tumour volumes No apparent adverse effects	This study also shows that SIRT1 stimulates the proliferation of endometrial carcinoma cells	Asaka et al.^55^
Mice	Pancreatic cancer model with PANC-1 xenograft	At 10 mg/kg i.p. alone, promotes the tumour growth No synergic effect with gemcitabine (however, almost no tumour growth was observed with gemcitabine alone)	–	Oon et al.^77^
Mice	Model of depression induced by chronic social defeat stress procedure	Injection in the nucleus accumbens at 0.5 µg/d blocks anxiety-like (open field, elevated maze) and social avoidance behaviours	BDNF signalling	Kim et al.^78^
Mice	Model of Parkinson’s disease (PD) induced by MPTP	At 10 mg/kg/d i.p., blocks the protective effects of resveratrol (which ameliorates the motor deficit and physiopathological changes)	Reduces SIRT1-mediated (activated by resveratrol) LC3 deacetylation and subsequent autophagic degradation of α-synuclein	Guo et al.^79^
Mice	Lung cancer model with A549 cells xenografts	At 30 mg/kg/d: Synergistically represses lung cancer growth with MK-1775 (WEE1 kinase inhibitor) No apparent toxicity on normal tissues	Reduces homologous recombination (HR) repair activity by acetylation of machinery proteins NBS1 and Rad51	Chen et al.^60^
Male Balb/C mice	Acute lung injury associated to endotoxemia, induced by LPS exposition	At 10 mg/kg, suppressed LPS-induced elevation of TNF-α and IL-6, and attenuated histological abnormalities	The beneficial effects were reversed by addition of an mTOR activator	Huang et al.^80^
Mice (ApoE^−/−^)	Atherosclerosis induced by collar placement around the carotid artery	At 10 mg/kg i.p., increases the atherosclerotic lesion	Decreases the autophagy process and enhances intraplaque macrophage infiltration	Yang et al.^61^
Mice (db/db)	Diabetic wound healing on diabetic mice	At 10 µM (topical application), delays diabetic wound healing promoted by resveratrol	Foxo1 and c-Myc transcription factors involved	Huang et al.^67^
Balb/C and several other mice	Graft-*versus*-host disease (GVHD) after mismatch grafts, and graft-*versus* leukaemia (GVL) treatment	At 2 mg/kg/d i.p., improves the clinical scores and prolongs survival in GVHD. Preserves the beneficial effect of graft in GVL treatment	Reduces T cell proliferation Less pathogenic T cells are generated Reduces pro-inflammatory cytokines production	Daenthanasanmak et al.^69^
Male Sprague-Dawley rats	Food intake of fasted animals	At 5 µg twice daily i.c.v. injection, decreases food intake and reduces body weight	Involvement of melanocortin receptors through SIRT1 mediated FoxO1 activity regulation	Çakir et al.^81^
Male Sprague-Dawley rats	Orexigenic action of ghrelin (food intake)	At 1 µg/rat i.c.v., decreased the orexigenic action of ghrelin	Blocks the activation of hypothalamic AMPK by ghrelin through p53 pathway (does not block the GH release)	Velásquez et al.^82^
Male Sprague-Dawley rats	Model of cerebral oxidative stress by intrastriatal infusion of malonate	At 1 µg (cerebrospinal concentration of ∼6 µM) reverses the beneficial effects (neurological improvement and reduction of striatal lesion) of PARP inhibition by 3-aminobenzamide	No effect on the neurological score and lesion when used alone (without 3-aminobenzamide)	Gueguen et al.^36^
Male Sprague-Dawley rats	Light-induced retinal damage	At 10 µg intravitreal injection, reduces the retinal protection by hydrogen-rich saline	Targets SIRT1 inhibition of apoptosis (through Bax and Bcl-2) and oxidative stress (through SOD)	Qi et al.^83^
Sprague-Dawley rats	Compression-induced skeletal muscle injury	At 1 mg/mg i.p., abolishes the protective effect of unacetylated ghrelin	Increases the levels of apoptosis and necroptosis in compressed muscle tissues despite the presence of unacetylated ghrelin	Ugwu et al.^84^
Male Sprague-Dawley rats	Model of partial hepatic warm ischaemia/reperfusion injury (microvascular clamp)	At 5 mg/kg i.v., decreases the beneficial effects on liver injury of a carbon monoxide-releasing molecule [Ru(CO)_3_Cl_2_]_2_	Decreases the inhibition of acetylation, translocation to the cytoplasm, and release of HMGB1 by [Ru(CO)_3_Cl_2_]_2_	Sun et al.^62^
Male Wistar rats	MCAO model of cerebral ischaemia	At 10 µg i.c.v., reduces the infarction volume of ischaemic brains and improves the survival (but not the neurological deficits)	Decreases *rip3* and *mlkl* gene expression and protein levels (regulators of necroptosis)	Nikseresht et al.^85^
Male Sprague-Dawley rats	Model of myocardial ischaemia/reperfusion injury	At 5 mg/kg/d i.p.: Abolished the beneficial effects of punicalagin (enhanced cardiac function and reduced myocardial infarction) No effect when administered alone on sham-operated rats	Blocks the beneficial effects of punicalagin on oxidative/nitrosative damage and inflammation, and reverses its activation of the NRF-2-HO-1 pathway	Yu et al.^86^
HD patients	HD	At doses up to 100 mg/d for 14 d, no observable clinical effects and no change in immune markers	No effect on levels of total circulating mHtt	Süssmuth et al.^73^

^a^ Sir2 is the homologue of mammalian SIRT1.

AMPK: AMP-activated protein kinase; ApoE: apolipoprotein E; BDNF: brain-derived neurotrophic factor; FoxO: forkhead box class O; Foxp3: forkhead box P3; HHUA and HEC1B: human endometrial carcinoma cells; HMGB1: high-mobility group box 1; HO-1: haem oxygenase-1; i.c.v.: intracerebroventricular; i.p.: intraperitoneal; LC3: microtubule-associated protein 1 light chain 3; LPS: lipopolysaccharides; MCAO: middle cerebral artery occlusion; mHtt: mutated Htt (huntingtin) exon 1 fragment with expanded Q repeat, presenting aggregates and cytotoxicity, model of Huntington’s disease; mlkl: mixed lineage kinase domain-like protein; MPTP: 1-methyl-4-phenyl-1, 2, 3, 6-tetrahydropyridine; mTOR: mammalian target of rapamycin; NRF-2: nuclear factor erythroid 2-related factor 2; PABPN1: polyadenylate-binding protein, nuclear 1; rip3: receptor-interacting protein kinase 3; Th17: T helper 17 cells (not naïve CD4 T cells); TNF-α: tumour necrosis factor-α; Tregs: T regulatory cells.

Pharmacokinetic data were obtained in mice and human, both in female and male. Selected parameters are given in Table 4. In R6/2 mice model of Huntington’s disease (HD) with 10–20 mg/kg dosing, average plasma concentrations over 24 h were in the low micromolar range (1.5–3.2 µM)^47^. In healthy male human volunteers with 150–300 mg doses, average plasma concentrations over 24 h were also in the low micromolar range (1.6–3.9 µM)^37^. However, a higher than proportional concentration (11.8 µM) was observed with 600 mg dosing, suggesting that one or more clearance mechanisms are approaching saturation at this dose. For multiple oral doses (for ex. 300 mg daily for 7 d for male), the data suggested that the pharmacokinetic steady-state was reached within 4 d, with an exposure higher than predicted from single-dose data.

The fraction of unchanged EX-527 excreted in the urine was very low for all doses in male subjects (<0.02% up to 24 h post-dose). The compound was transformed *in vivo* by hydroxylation and oxidative deamination followed by glucuronic acid conjugation, across all species studied (mouse, rat, dog, and human)^37^.

Pharmacogenomics studies suggested that EX-527 treatment in human was associated with a specific transcriptional signature in blood cells, with genes involved in mechanisms of signal transduction and transmembrane transport, as well as metabolic and redox processes^37^.

The conclusion of the safety study in healthy volunteers indicated that EX-527 was safe and well tolerated by female and male subjects after single doses up to 600 mg and multiple doses up to 300 00/d for 7 d. Moreover, no meaningful cardiovascular effects were observed in beagle dogs up to 100 mg/kg^37^.

*In vivo*, numerous studies have been carried out to explore the effect of EX-527 under physiological or pathological conditions (see Table 5 for representative examples). Although most cell-based assays used cancer cells, *in vivo*, EX-527 was assayed in a more diverse set of pathologies, and only in a small number of cancer models on mice xenograft. Overall, it appeared very well tolerated when administered alone, in agreement with the phase I clinical trial described above^37^.

Apparent detrimental effects of EX-527 often consisted in inhibition of beneficial effects induced by additional compounds. For example, mice and rats suffering from ischaemia, sepsis, or chronic obstructive pulmonary disease were treated with several natural products including melatonin^76^^,^^87–89^, diallyl trisulphide^90^, and punicalagin^86^. Other examples include the effects of ghrelin^82^^,^^84^, hydrogen-rich saline^83^, carbon monoxide^62^, the SIRT1 activators resveratrol^67^^,^^79^^,^^91^ and scopolin^92^, and the PARP inhibitor 3-aminobenzamide^36^. In all these cases, EX-527 was used as a pharmacological tool to demonstrate that SIRT1 activation was involved in the beneficial effects of the compounds under study. When used alone, a detrimental effect of EX-527 on pancreatic tumour xenograft was observed in one study, which gave surprising results^77^. Indeed, EX-527 increased the cytotoxic effect of gemcitabine *in vitro* in PANC-1 cells, in agreement with another study^50^, but it activated the tumour xenograft of the same cells *in vivo*^77^. The activity of EX-527 on other cell types in the tumour microenvironment is a possible explanation for this discrepancy. We note that in this xenograft study, the addition of EX-527 at 10 mg/kg with gemcitabine apparently did not have any effect, but the tumour growth in the control experiments with gemcitabine alone was already very limited.

Beneficial effects were observed in several pathologies. In cancer, EX-527 decreased the tumour growth of xenografted mice with endometrial and lung cancer cells^60^^,^^55^. In immunity-related diseases, a first report in 2011 indicated that, when used in combination with rapamycin, it prolonged heart allograft survival in mice^74^. The involvement of Tregs through increased expression of Foxp3 was proposed. Other studies confirmed these beneficial effects of EX-527 on Tregs through increased Foxp3 expression and acetylation, and the possible involvement of another SIRT1 substrate, NF-κB^69^^,^^75^^,^^93^. In a mouse model of multiple sclerosis, an immune disorder, it strongly suppressed the number of paralysed mice, through an effect of Th17 effector cells^57^.

In a phase II clinical trial involving HD patients, EX-527 was found to be safe and well-tolerated^73^. However, no clinical benefit was observed after the two weeks treatment. For this slowly progressive neurodegenerative disease, longer treatment durations of 2 years may be required to observe clinical benefits. In addition, and maybe for the same reason, no effects on the levels of soluble mutated huntingtin (mHtt) in healthy peripheral blood mononuclear cells (PBMCs) were observed.

## 5. Conclusion

EX-527 has been tested on many cell lines, alone or in combination with other molecules, resulting in a variety of cellular effects. Moreover, it displayed several biological effects *in vivo* in various pathological conditions. These results are in agreement with the fact that its specific target SIRT1 is a key regulator of cell fate, through its deacetylation action on a large number of protein substrates. The expression and the activity of SIRT1 can be either up- or down-regulated, depending on the cellular state in the physiological or pathological conditions under study. The administration of EX-527 appears to be beneficial in cases where the activity of SIRT1 is upregulated. Perhaps the most promising *in vivo* results have been obtained on mice and rats in autoimmune diseases and allograft tolerance, with a significant increase in survival.

Although the results of a phase II clinical trial in HD did not provide the expected beneficial effects, the safety of EX-527 was demonstrated with patients in phase I clinical trials. Therefore, further preclinical and clinical studies in other pathologies appear attractive. In this way, the SIRT1 Antagonism For Endometrial Receptivity (SAFER) clinical trial with EX-527 (Selisistat) will enrol around 30 women with unexplained failure after embryo transfer with euploid embryos. This phase II trial will start on 1 January 2021, and finish on 31 December 2022. The drug will be administered daily for 5 d, beginning with the start of progesterone therapy, and ending 24 h before embryo transfer. Pregnancy rates and pregnancy outcome will be monitored (trial number NCT04184323).

New derivatives of EX-527 with greater activity and selectivity for SIRT1, as well as improved pharmacokinetic and pharmacodynamic properties, may lead to results that are even more promising, and reach further advanced clinical trials.
